# Commentary: Some Social, Psychological, and Political Factors That Undermine Compliance With COVID-19 Public Health Measures

**DOI:** 10.3389/ijph.2021.603944

**Published:** 2021-03-12

**Authors:** Kathleen D. Magnus

**Affiliations:** University Medical Center Hamburg-Eppendorf, Hamburg, Germany

**Keywords:** COVID-19, right-wing populism, cognitive bias, compliance, public health restrictions, groupthink

Mask-wearing, social distancing, and hand hygiene have proven effective in mitigating the spread of the COVID-19 virus. However, despite widespread campaigns to inform the public, compliance with these measures has been less than optimal in many Western countries. Various groups, including anti-vaxxers, conspiracy theorists, self-proclaimed “free thinkers,” and far-right extremists, have protested against public health regulations designed to contain the COVID-19 virus. Unlike the opposition to lockdowns, which is often based on competing rational concerns, the resistance to simple practices like mask-wearing defies scientific reason. Insufficient or false information may explain some of this resistance, but there are a number of psychological, social, and political factors that contribute to it as well.

In what follows, I briefly discuss some of the cognitive biases and social dynamics which may lead people to oppose public health efforts to reduce the spread of the COVID-19 virus. In addition, I draw attention to how these psychological and social tendencies have been exploited by politically motivated groups, especially right-wing populists who stand to benefit when people suffer poor health (1, 2). Although public health officials may not be able to intervene directly in the political sphere, they can take some steps to increase compliance with COVID-19 mitigating measures and to counter the political forces that seek to undermine them.

A number of cognitive biases may lead even the most reasonable people to disregard public health recommendations at least some of the time. For example, a particularly prevalent bias, the “optimism bias,” may incline people to underestimate their own chances of getting sick (3). In other words, even people who recognize the real risk of catching COVID-19 may maintain a strong belief that *they* will not catch it. (“Sure, it’s a problem, but *I* won’t get sick.”) In some cases, this belief is so strong that people neglect to wear a mask or to social distance even when these precautions are clearly warranted. This bias may come into play regardless of an individual’s social situation or political affiliations; however, it becomes particularly problematic when combined with the exceptionalism promulgated by certain right-wing populist groups. By encouraging people to see themselves as belonging to a special, “protected” group, right-wing populist leaders transform the individual defense mechanism (“*I* won’t get sick”) into an issue of greater social relevance (“it’s not our problem,” “it’s an urban issue,” etc.). This happened in the United States last spring. After right-wing populist media propagated the idea that the COVID-19 virus was “a city problem,” many local and state governments failed to implement adequate measures to prevent the spread of the virus in rural areas (4).

The situation is similar with respect to the “normalcy bias.” Given a strong psychological preference for normalcy, people are reluctant to prepare for disaster scenarios or to respond to them when they occur (5). This bias explains why some people refuse to evacuate their homes when authorities warn them of approaching hurricanes, floods, or forest fires, and in the context of the current health crisis, it may also explain the reluctance some people have to protect themselves against COVID-19. Again this bias may affect any individual regardless of social situation or political affiliations; however, it plays especially well into the hands of right-wing populist leaders and conspiracy theorists who want to convince people that the virus poses no serious threat. Because people are already inclined to doubt that abrupt, disastrous change can happen, many are quick to follow right-wing populist leaders who downplay the virus and flout the advice of public health experts.

Another reason people may choose to ignore public health regulations stems from the fundamentally social nature of the human psyche. People develop their identities in relation to others, and their own judgments are heavily influenced by what others think (6). Moreover, the desire to belong to the “in-group” generates a will to conform that often leads to false conclusions (7), and this desire has been shown to influence people’s decisions about their own health (8). In some societies this tendency to conform has translated into a unified response to mitigate the spread of COVID-19, but in other countries it has been used to undermine measures that are meant to reduce contagion. Right-wing populists in the United States and Europe serve as prime examples of this dynamic. By turning the refusal to wear masks into a symbol of group identity, they undermine health experts, encourage citizens to risk their health, and stoke social division all at once.

The public health community may counter these and other psychological, social, and political forces through short and long-term initiatives. Clear, consistent, fact-based information goes a long way to keep irrational judgments and behaviors in check. However, given the deluge of misinformation that circulates through various media outlets, public health information campaigns must be vigorous and unrelenting. Even so, they will not suffice to change the behavior of people who are committed to their own cognitive biases and deeply invested in the belief that the opinions of their group are superior to all others. For this reason, long-term programs that teach people how to recognize and reflect upon their own cognitive biases and psychological vulnerabilities must be developed. In the meantime, public health officials must acknowledge the powerful social, psychological, and political forces that undermine compliance, especially those stemming from right-wing populists who have a long-standing record of opposing public health measures (9, 10). Moreover, given the urgency of the current health crisis, officials should not hesitate to implement regulations with harsh penalties for non-compliance. This may be the only way to avoid more severe restrictions such as lockdowns, which are bound to provoke even greater resistance.
